# Supplementary material for: Lived experiences of people living with HIV—A qualitative exploration on the manifestation, drivers, and effects of internalized HIV stigma within the Malawian context
Source: PLoS One. 2023 Apr 27;18(4):e0284195. doi: 10.1371/journal.pone.0284195 (PMC10138249; doi:10.1371/journal.pone.0284195)
Supplement: S1 Appendix — (DOCX) [file pone.0284195.s001.docx]

**Appendix 1: Data Collection tools**

**Appendix 1a: Focus Group Discussion Topic guide**

**Introduction:**

Good day. My name is………………………Am here on behalf of the International AIDS Society and … (Local institution that will support us eg MANET as appropriate) conducting a study on understanding stigma in Malawi in order to improve the services this facility and others offer and would like to ask you some questions about your experiences. We would like your personal opinion on various health services in Malawi. There are five parts to this interview and it should take about 60 minutes to complete.

Your personal observations and perceptions are anonymous and you will not be associated with them because they will be entered into a large database with many other opinions. We will only ask your sex and age.

Participating in this research is voluntary. You will not receive any compensation for participating. You will also not be punished if you decline to participate. At any time you can refuse to answer a question or stop participating altogether without any penalty.

Do you have any questions for me? Do I have your permission to continue with the interview?

|  |  |  |  |  | **2** | **0** | **2** | **1** |
| --- | --- | --- | --- | --- | --- | --- | --- | --- |
| Interviewer's signature  (Indicates respondent's willingness to participate) | DAY | | MONTH | | YEAR | | | |

Many thanks for agreeing to take part in this group discussion. As you know HIV-related stigma is a main issue that has affected the access and delivery of HIV care in Malawi. We are implementing a study that is aimed on getting to the heart of stigma by conducting in-depth analyses of the country situation and recent improvements, policies and research to generate a vivid picture of lived experiences of people when they encounter HIV-related stigma. Stigma is grouped into three categories: external stigma which is stigmatizing behavior from others directed toward PLHIV; internal stigma internalizes the devaluation from others whereby the PLHIV stigmatizes self; and stigma by association (secondary) whereby those associated with PLHIV are stigmatized such as family members and friends. By speaking to this group today we hope to learn more about the nature and manifestation of stigma within Malawi while drawing out best practices on interventions aimed at addressing this problem. We would first like to begin by knowing you and what you do.

**Experiences of HIV Stigma**

We would now want to know about personal or witnessed episode of HIV stigma in your community, health facilities or work places.

- Have you or someone that you know experienced social stigma associated with HIV? If yes, please describe what happened? *(Probe: How did you feel about this incident that happened to you? How did you handle the situation? If it was someone, how did the stigmatised person act? How did that someone or family member handle the situation?)*

**Establishing evidence on local innovations for stigma reduction.**

We would now like to better understand local practices that have been used to reduce stigma linked to HIV.

- Please can you walk me through all the different approaches that have been used to reduce stigma in Malawi. *(Probe: How have these interventions been implemented and where? Who led the implementation? Who participated in the implementation? What were the target groups for such interventions? Which approaches have been successful and why? Which approaches have not been successful at reducing stigma and why?)*
- What are the local interventions or innovations that were developed to specifically address the stigma situation in Malawi? (Probe: Which of these are promising and why? Which of the interventions have not been documented?)

**Inspiration stories on Stigma reduction in Malawi.**

Let us now discuss stories of interventions on stigma reduction that some healthcare workers have done in order to reduce HIV stigma at their health facility or in the community.

- Please could you tell me any interesting story that you know regarding what one or several health workers have done in order to reduce stigma in their facility or community. *(Probe: please explain in detail the sequence of events that led to this? What were the motivations of healthcare workers to do this? What challenges did they experience as they were implementing this? What support was needed or was provided for them to do this? How did this reduce HIV stigma at the facility or in the community? Did it change social norms and behaviors around social stigma?)*
- How do you think this intervention/s compares to the usual way of reducing HIV-related stigma?

*(Probe: please indicate these differences when compared to the usual approaches)*

- Did the implementation of such effort went according to plan? *(Probe: Were there any unexpected things that were experienced or came out of the process of introducing such interventions?)*
- Were the key lessons learned from this shared with other? (Probe: Were there other facilities that emulated this?
- Have the intervention changed local practices or national policy on stigma reduction? (Probe: when thinking back to these changes, can you explain any successes and challenges that were experienced?)

**Suggestions for reducing normalized social stigma**

As we are about to conclude our conservation, I would like to know if you have some ideas on how to reduce HIV stigma.

- What do you suggest need to be done to reduce self stigma?
- What do you think can be practical solutions to the issue of HIV-related stigma in Malawi ?

We have reached the end of our talk. Thank you very much for your time.

**Appendix 1b: Key Informant Interview Topic guide (PLHA/KP/AGYW)**

**Introduction:**

Good day. My name is………………………Am here on behalf of the International AIDS Society and … (Local institution that will support us eg MANET as appropriate) conducting a study on understanding stigma in Malawi in order to improve the services this facility and others offer and would like to ask you some questions about your experiences. We would like your personal opinion on various health services in Malawi. There are five parts to this interview and it should take about 60 minutes to complete.

Your personal observations and perceptions are anonymous and you will not be associated with them because they will be entered into a large database with many other opinions. We will only ask your sex and age.

Participating in this research is voluntary. You will not receive any compensation for participating. You will also not be punished if you decline to participate. At any time you can refuse to answer a question or stop participating altogether without any penalty.

Do you have any questions for me? Do I have your permission to continue with the interview?

|  |  |  |  |  | **2** | **0** | **2** | **1** |
| --- | --- | --- | --- | --- | --- | --- | --- | --- |
| Interviewer's signature  (Indicates respondent's willingness to participate) | DAY | | MONTH | | YEAR | | | |

Many thanks for agreeing to take part in this interview. As you know HIV-related stigma is a main issue that has affected the access and delivery of HIV care in Malawi. We are implementing a study that is aimed on getting to the heart of stigma by conducting in-depth analyses of the country situation and recent improvements, policies and research to generate a vivid picture of lived experiences of people when they encounter HIV-related stigma. By speaking to you today we hope to learn more about the nature and manifestation of stigma within Malawi while drawing out best practices on interventions aimed at addressing this problem. We would first like to begin by knowing you and what you do.

**Participants’ details:**

We would first like to begin by getting to know more about you.

- Please could you tell us more about yourself. What do you do?
- What does a normal day look like? How long have you had this occupation? (*Probes: How long have you been doing this? What were you doing before?)*
- What did you feel about HIV before knowing your status? (Probe: why did you feel that way?)
- How do you feel about HIV after knowing that you have HIV?

**HIV Sero-status disclosure**

- What do you think about an individual sharing their HIV+ status with other people?
- Did you share to anybody about your status after learning that you have HIV? (Probe: Provide reasons for sharing or not sharing your HIV+ status)
- **For those that disclosed;** describe how you shared this information and to whom? (*Probe: What did you feel about your experiences after letting others know about your HIV+? Was this the right thing to do?)*
- Based on your experiences, what do you think is the most difficult thing for a person living with HIV to do? Why?

**Access to treatment and psychosocial support**

- Why do you think some people living with HIV travel to distant places other than the clinic closest to them for ART? *(Probe: Do you know people who have done this? If yes, why do you think they do this? Have you done it before? Why?)*
- Have you ever shared with anybody about your inner feelings about being HIV+? (*Probe: What did you share? How easy or difficult was it to share? if yes, what was the advice that was given to you? Was it helpful to share your inner feelings with someone? If no, do you feel like talking with someone to share your inner feelings? Who would you want to share these feelings with and why?)*
- Has anybody asked you to share what you feel about living with HIV? If yes who was it? What did you think about somebody asking you? If not what would you share if somebody asked you?
- Have you ever felt helpless because of being HIV+? If yes can you describe what made you to feel that way? what did you do about it?

**Perception about stigma**

- Are people living with HIV different from others? *(Probe: Explain your answer. Is there any difference between people living with HIV in the community when compared to people living with HIV who are also sex workers or men who have sex with men or the youth or the elderly?)*
- Do people living with HIV have personal worthiness/value? Explain your answer. *(Probe: In relation to personal value or worthness is there any difference between people living with HIV in the community compared to people living with HIV who are also sex workers or men who have sex with men or the youth or the elderly)*
- Do you know other people living with HIV who feel unworthy/valueless, helpless? *(Probe: Have you ever helped such people? If yes how? If no, why?)*
- Do people living with HIV need preferential treatment in the home, school, workplace, church, community, hospital? Explain your answer *(Probe: Should there be any difference in providing treatment to people living with HIV in the community when compared to people living with HIV who are also sex workers or men who have sex with men or the youth or the elderly)*
- How do issues such as age, ethnicity, culture, sex, religious affiliation influence self-stigma among people living with HIV? *(Probe: Does one’s education background influence self stigma? Why and how?)*
- Do you know of individuals, institutions which help such people? If yes can you explain what kind of help/support? Have these institutions provided different forms of support to other people living with HIV such as sex workers or men who have sex with men or the youth or the elderly)

**Experience of stigma**

- Have you experienced social stigma associated with HIV? If yes, please describe what happened? *(Probe: How did you feel about this incident? How did you handle it? How did the stigmatised person act if it was not you personally? if the stigma happened to another person, how could you have handled the situation if it happened to you or your family member?)*
- How do incidences of self-stigma handled by the affected individuals? What about you, how do you handle feelings of self stigmatization?
- How does the community handle self-stigma? Do you think what is being done currently is adequate in addressing self-stigma in your community?
- What are the problems that come because of self-stigma? *(Probe: can you give example of people who are experiencing these problems)*
- How can we best address the problem of self-stigma? (Probe: Can you suggest ways of addressing this? Who is best suited to address self-stigma as individual, institution etc?)

We have reached the end of our talk. Thank you very much for your time.

**Appendix 1c: Key Informant Interview Topic guide (ART Providers & HTC providers)**

**Introduction:**

Good day. My name is………………………Am here on behalf of the International AIDS Society and … (Local institution that will support us eg MANET as appropriate) conducting a study on understanding stigma in Malawi in order to improve the services this facility and others offer and would like to ask you some questions about your experiences. We would like your personal opinion on various health services in Malawi. There are five parts to this interview and it should take about 60 minutes to complete.

Your personal observations and perceptions are anonymous and you will not be associated with them because they will be entered into a large database with many other opinions. We will only ask your sex and age.

Participating in this research is voluntary. You will not receive any compensation for participating. You will also not be punished if you decline to participate. At any time you can refuse to answer a question or stop participating altogether without any penalty.

Do you have any questions for me? Do I have your permission to continue with the interview?

|  |  |  |  |  | **2** | **0** | **2** | **1** |
| --- | --- | --- | --- | --- | --- | --- | --- | --- |
| Interviewer's signature  (Indicates respondent's willingness to participate) | DAY | | MONTH | | YEAR | | | |

Many thanks for agreeing to take part in this interview. If you are comfortable we will now begin. As you know HIV-related stigma is a main issue that has affected the access and delivery of HIV care in Malawi. We are implementing a study that is aimed on getting to the heart of stigma by conducting in-depth analyses of the country situation and recent improvements, policies and research to generate a vivid picture of lived experiences of people when they encounter HIV-related stigma. By speaking to you today we hope to learn more about the nature and manifestation of stigma within Malawi while drawing out best practices on interventions aimed at addressing this problem. We would first like to begin by knowing you and what you do.

**Participants’ details:**

We would first like to begin by getting to know more about you.

- Please could you tell us more about yourself, position. What do you do?
- What does a normal day look like? How long have you had this occupation? (*Probes: How long have you been doing this? What were you doing before?)*
- What do you think is the current situation regarding stigma in Malawi *(Probe: How is a situation for HIV infected individuals, what about families affected with HIV, how has this changed over time?)*
- How does stigma situation in Malawi affect access to HIV services? (*Probe: which groups are greatly affected by this and why, Are there any differences in HIV stigma between children, youth and adult men and women? )*
- How does the stigma situation affect health delivery of HIV-related services?

**Experiences of HIV Stigma**

We would now want to know about personal or witnessed episode of HIV stigma.

- Have you or someone that you know experienced social stigma associated with HIV? If yes, please describe what happened? *(Probe: How did you feel about this incident, how did the stigmatised person act? if the stigma happened to another person, how could you have handled the situation if it happened to you or your family member?)*
- Do you think self-stigma exists among PLHIV or Key populations or Youth or the Elderly? Explain. (Probe: Who do you think experience self-stigma most? Why?)
- How does issues such as age, ethnicity, culture, sex, religious affiliation influence self-stigma among people living with HIV?
- Have you seen some people living with HIV/KPs travelling to distant facilities to access HIV services eg testing or ART that is available in their area? (Probe: Why do you think they do this?)
- What form of support is given to a stigmatised individual by the people who were present when this happened?
- **For HCW who live with HIV:** How does your HIV status influence provision of care to PLHIV? How does it increase or reduce self stigma?

**Establishing evidence on local innovations for stigma reduction.**

We would now like to better understand local practices that have been used to reduce stigma linked to HIV.

- What national policy guidance is available to address the problem of HIV stigma in Malawi? *(Probe: How are these policies implemented? What are the gaps in policy implementation around HIV stigma reduction? Are you aware of the HIV Management Act? If yes, how does it help reduce stigma in health care settings?)*
- Please can you walk me through all the different approaches that have been used to reduce stigma in Malawi. *(Probe: How have these interventions been implemented and where? Who led the implementation? Who participated in the implementation? What were the target groups for such interventions? Which approaches have been successful and why? Which approaches have not been successful at reducing stigma and why?)*
- What are the local interventions or innovations that were developed to specifically address the stigma situation in Malawi? (Probe: Which of these are promising and why? Which of the interventions have not been documented? Which of these are specifically for reducing self-stigma?)
- Are there healthcare providers at your facilities who have been trained to handle people with self-stigma? *(Probe: Have you been trained? Are there materials/equipment/treatment and space for privacy in handling such people? Is what you do supported by policy, law? Explain?)*
- How do you report and manage cases of self-stigma? (Probe: Are you satisfied with current situation in addressing self-stigma? What do you suggest should happen to improve the situation?)
- How is success defined when dealing with self-stigma in Malawi?

**Inspiration stories on Stigma reduction in Malawi.**

Let us now discuss stories of interventions on stigma reduction that some healthcare workers have done in order to reduce HIV stigma at their health facility or in the community.

- Please could you tell me any interesting story that you know regarding what one or several health workers have done in order to reduce stigma in their facility or community. *(Probe: please explain in detail the sequence of events that led to this? What were the motivations of healthcare workers to do this? What challenges did they experience as they were implementing this? What support was needed or was provided for them to do this? How did this reduce HIV stigma at the facility or in the community? Did it change social norms and behaviours around social stigma?)*
- How do you think this intervention/s compares to the usual way of reducing HIV-related stigma? *(Probe: please indicate these differences when compared to the usual approaches)*
- As you think back to how this was done, do you remember if this went to plan? *(Probe: Were there any unexpected things that were experienced or came out of the process of introducing such interventions?)*
- What was done to ensure that lessons learned from this story were maintained and shared? (Probe: Were there other facilities that emulated this?
- Have the interventions changed local practices or national policy on stigma reduction? (Probe: when thinking back to these changes, can you explain any successes and challenges that were experienced?)

**Suggestions for reducing normalised social stigma**

As we are about to conclude our conservation, I would like to know if you have some ideas on how to reduce HIV stigma.

- What do you suggest need to be done to reduce self stigma?
- What do you think can be practical solutions to the issue of HIV-related stigma in Malawi

We have reached the end of our talk. Thank you very much for your time.

**Appendix 1d: Key Informant Interview Topic guide**

**Introduction:**

Good day. My name is………………………Am here on behalf of the International AIDS Society and … (Local institution that will support us eg MANET as appropriate) conducting a study on understanding stigma in Malawi in order to improve the services this facility and others offer and would like to ask you some questions about your experiences. We would like your personal opinion on various health services in Malawi. There are five parts to this interview and it should take about 60 minutes to complete.

Your personal observations and perceptions are anonymous and you will not be associated with them because they will be entered into a large database with many other opinions. We will only ask your sex and age.

Participating in this research is voluntary. You will not receive any compensation for participating. You will also not be punished if you decline to participate. At any time you can refuse to answer a question or stop participating altogether without any penalty.

Do you have any questions for me? Do I have your permission to continue with the interview?

|  |  |  |  |  | **2** | **0** | **2** | **1** |
| --- | --- | --- | --- | --- | --- | --- | --- | --- |
| Interviewer's signature  (Indicates respondent's willingness to participate) | DAY | | MONTH | | YEAR | | | |

Many thanks for agreeing to take part in this interview. As you know HIV-related stigma is a main issue that has affected the access and delivery of HIV care in Malawi. We are implementing a study that is aimed on getting to the heart of stigma by conducting in-depth analyses of the country situation and recent improvements, policies and research to generate a vivid picture of lived experiences of people when they encounter HIV-related stigma. By speaking to you today we hope to learn more about the nature and manifestation of stigma within Malawi while drawing out best practices on interventions aimed at addressing this problem. We would first like to begin by knowing you and what you do.

**Participants’ details:**

We would first like to begin by getting to know more about you.

- Please could you tell us more about yourself, position? What do you do?
- What does a normal day look like? How long have you had this occupation? (*Probes: How long have you been doing this? What were you doing before?)*
- What is the current situation regarding stigma in Malawi *(Probe: How is a situation for HIV infected individuals, what about families affected with HIV, how has this changed over time?)*
- How does stigma situation in Malawi affect access to HIV services? (*Probe: which groups are greatly affected by this and why, Are there any differences in HIV stigma between children, youth and adult men and women? )*
- How does the stigma situation affect delivery of HIV-related services?

**Experiences of HIV Stigma**

We would now want to know about personal or witnessed episode of HIV stigma.

- Do you think self-stigma exists among PLHIV or Key populations or Youth or the Elderly? Explain. (Probe: Who do you think experience self-stigma most? Why?)
- How do issues such as age, ethnicity, culture, sex, religious affiliation influence self-stigma among people living with HIV?
- What problems do you know that come as a result of self-stigma?
- Have you seen some people living with HIV/KPs travelling to distant facilities to access HIV services eg testing or ART that is available in their area? *(Probe: Why do you think they do this? What do you think needs to be done?)*
- What form of support is given to a stigmatised individual by the people who were present when this happened?

**Establishing evidence on local innovations for stigma reduction.**

We would now like to better understand local practices that have been used to reduce stigma linked to HIV.

- What national laws, policies and guidelines are available to address the problem of HIV stigma in Malawi? *(Probe: How are these policies implemented? What are the gaps in policy implementation around HIV stigma reduction? What would it take to have policies, guidelines and laws in place? Do the existing laws and policies address self-stigma? How? If not, what do you suggest need to be done? How?)*
- Please can you walk me through all the different approaches that have been used to reduce stigma in Malawi. *(Probe: How have these interventions been implemented and where? Who led the implementation? Who participated in the implementation? What were the target groups for such interventions? Which approaches have been successful and why? Which approaches have not been successful at reducing stigma and why? Is there training for handling self-stigma?)*

1. What are the local interventions or innovations that were developed to specifically address the stigma situation in Malawi? *(Probe: Which of these are promising and why? Which of the interventions have not been documented? Are there material/equipment/treatment provision including space for privacy in handling people with self-stigma?)*

- Are there healthcare providers at your facilities who have been trained to handle people with self-stigma? (Probe: Have you been trained? Are there materials/equipment/treatment and space for privacy in handling such people? Is what you do supported by policy, law? Explain?)
- How do you report and manage cases of self-stigma? *(Probe: Are you satisfied with current situation in addressing self-stigma? What do you suggest should happen to improve the situation? Are there current practices in dealing with self-stigma supported by policy, guidelines and law? )*
- How is success defined when dealing with self-stigma in Malawi?

**Inspiration stories on Stigma reduction in Malawi.**

Let us now discuss stories of interventions on stigma reduction that some healthcare workers have done in order to reduce HIV stigma at their health facility or in the community.

- Please could you tell me any interesting story that you know regarding what one or several health workers have done in order to reduce stigma in their facility or community. *(Probe: please explain in detail the sequence of events that led to this? What were the motivations of healthcare workers to do this? What challenges did they experience as they were implementing this? What support was needed or was provided for them to do this? How did this reduce HIV stigma at the facility or in the community? Did it change social norms and behaviours around social stigma?)*
- How do you think this intervention/s compares to the usual way of reducing HIV-related stigma?

*(Probe: please indicate these differences when compared to the usual approaches)*

- As you think back to how this was done, do you remember if this went to plan? *(Probe: Were there any unexpected things that were experienced or came out of the process of introducing such interventions?)*
- What was done to ensure that lessons learned from this story were maintained and shared? (Probe: Were there other facilities that emulated this?
- Have the interventions changed local practices or national policy on stigma reduction? (Probe: when thinking back to these changes, can you explain any successes and challenges that were experienced?)

**Suggestions for reducing normalised social stigma**

As we are about to conclude our conservation, I would like to know if you have some ideas on how to reduce HIV stigma.

- What do you suggest need to be done to reduce self stigma?
- What do you think can be practical solutions to the issue of HIV-related stigma in Malawi?

We have reached the end of our talk. Thank you very much for your time.

**Appendix 1e: Key Informant Interview Topic guide (Legal and Social Services provider)**

**Introduction:**

Good day. My name is………………………Am here on behalf of the International AIDS Society and … (Local institution that will support us eg MANET as appropriate) conducting a study on understanding stigma in Malawi in order to improve the services this facility and others offer and would like to ask you some questions about your experiences. We would like your personal opinion on various health services in Malawi. There are five parts to this interview and it should take about 60 minutes to complete.

Your personal observations and perceptions are anonymous and you will not be associated with them because they will be entered into a large database with many other opinions. We will only ask your sex and age.

Participating in this research is voluntary. You will not receive any compensation for participating. You will also not be punished if you decline to participate. At any time you can refuse to answer a question or stop participating altogether without any penalty.

Do you have any questions for me? Do I have your permission to continue with the interview?

|  |  |  |  |  | **2** | **0** | **2** | **1** |
| --- | --- | --- | --- | --- | --- | --- | --- | --- |
| Interviewer's signature  (Indicates respondent's willingness to participate) | DAY | | MONTH | | YEAR | | | |

Many thanks for agreeing to take part in this interview. If you are comfortable we will now begin. As you know HIV-related stigma is a main issue that has affected the access and delivery of HIV care in Malawi. We are implementing a study that is aimed on getting to the heart of stigma by conducting in-depth analyses of the country situation and recent improvements, policies and research to generate a vivid picture of lived experiences of people when they encounter HIV-related stigma. By speaking to you today we hope to learn more about the nature and manifestation of stigma within Malawi while drawing out best practices on interventions aimed at addressing this problem. We would first like to begin by knowing you and what you do.

**Participants’ details:**

We would first like to begin by getting to know more about you.

- Please could you tell us more about yourself, position. What do you do?
- What does a normal day look like? How long have you had this occupation? (*Probes: How long have you been doing this? What were you doing before?)*
- What do you think is the current situation regarding stigma in Malawi *(Probe: How is a situation for HIV infected individuals, what about families affected with HIV, how has this changed over time?)*
- How does stigma situation in Malawi affect access to HIV services? (*Probe: which groups are greatly affected by this and why, Are there any differences in HIV stigma between children, youth and adult men and women? )*
- How does the stigma situation affect health delivery of HIV-related services?

**Experiences of HIV Stigma**

We would now want to know about personal or witnessed episode of HIV stigma.

- Have you or someone that you know experienced social stigma associated with HIV? If yes, please describe what happened? *(Probe: How did you feel about this incident, how did the stigmatised person act? if the stigma happened to another person, how could you have handled the situation if it happened to you or your family member?)*
- Do you think self-stigma exists among PLHIV or Key populations or Youth or the Elderly? Explain. (Probe: Who do you think experience self-stigma most? Why?)
- How does issues such as age, ethnicity, culture, sex, religious affiliation influence self-stigma among people living with HIV?
- Have you seen some people living with HIV/KPs travelling to distant facilities to access HIV services eg testing or ART that is available in their area? (Probe: Why do you think they do this?)
- What form of support is given to a stigmatised individual by the people who were present when this happened?
- **For Police and social welfare officers who live with HIV:** How does your HIV status influence provision of care to stigmatised PLHIV? How does it increase or reduce self stigma?
- What problems do you know that comes as a result of self-stigma?

**Establishing evidence on local innovations for stigma reduction.**

We would now like to better understand local practices that have been used to reduce stigma linked to HIV.

- What national policy guidance is available to address the problem of HIV stigma in Malawi? *(Probe: How are these policies implemented? What are the gaps in policy implementation around HIV stigma reduction? Are you aware of the HIV Management Act? If yes, how does it help reduce stigma in health care settings?)*
- Please can you walk me through all the different approaches that have been used to reduce stigma in Malawi. *(Probe: How have these interventions been implemented and where? Who led the implementation? Who participated in the implementation? What were the target groups for such interventions? Which approaches have been successful and why? Which approaches have not been successful at reducing stigma and why?)*
- What are the local interventions or innovations that were developed to specifically address the stigma situation in Malawi? (Probe: Which of these are promising and why? Which of the interventions have not been documented? Which of these are specifically for reducing self-stigma?)
- Are there police officers/social welfare officers at your facilities who have been trained to handle people with self-stigma including HIV-related stigma? *(Probe: Have you been trained? Are there materials/equipment/treatment and space for privacy in handling such people? Is what you do supported by policy, law? Explain?)*
- Are there other individuals or institutions who support your work? How and why?
- How do you report and manage cases of self-stigma? (Probe: Are you satisfied with current situation in addressing self-stigma? What do you suggest should happen to improve the situation?)
- How is success defined when dealing with self-stigma in Malawi?

**Inspiration stories on Stigma reduction in Malawi.**

Let us now discuss stories of interventions on stigma reduction that some healthcare workers have done in order to reduce HIV stigma at their health facility or in the community.

- Please could you tell me any interesting story that you know regarding what one or several health workers have done in order to reduce stigma in their facility or community. *(Probe: please explain in detail the sequence of events that led to this? What were the motivations of healthcare workers to do this? What challenges did they experience as they were implementing this? What support was needed or was provided for them to do this? How did this reduce HIV stigma at the facility or in the community? Did it change social norms and behaviours around social stigma?)*
- How do you think this intervention/s compares to the usual way of reducing HIV-related stigma? *(Probe: please indicate these differences when compared to the usual approaches)*
- As you think back to how this was done, do you remember if this went to plan? *(Probe: Were there any unexpected things that were experienced or came out of the process of introducing such interventions?)*
- What was done to ensure that lessons learned from this story were maintained and shared? (Probe: Were there other facilities that emulated this?
- Have the interventions changed local practices or national policy on stigma reduction? (Probe: when thinking back to these changes, can you explain any successes and challenges that were experienced?)

**Suggestions for reducing normalised social stigma**

As we are about to conclude our conservation, I would like to know if you have some ideas on how to reduce HIV stigma.

- What do you suggest need to be done to reduce self stigma?
- What do you think can be practical solutions to the issue of HIV-related stigma in Malawi

We have reached the end of our talk. Thank you very much for your time.
