# Supplementary material for: Lived experiences of people living with HIV—A qualitative exploration on the manifestation, drivers, and effects of internalized HIV stigma within the Malawian context
Source: PLoS One. 2023 Apr 27;18(4):e0284195. doi: 10.1371/journal.pone.0284195 (PMC10138249; doi:10.1371/journal.pone.0284195)
Supplement: Attachment — Submitted filename: Comments to the Authors_cyk.docx [file pone.0284195.s003.docx]

**Comments to the Authors**

Dear authors,
Thank you for the chance to offer feedback. I thoroughly enjoyed your article and believe that paper is worth pursuing.
I have offered some feedback below. I hope you find my comments/suggestions helpful.

**INTRODUCTION**

- “More than a third of people living with HIV (PLHIV) report having been physically assaulted…” was written/reported in 2012.

As this is a prevalence report, better to provide more recent data/references.

- “A recent systematic review and meta-analysis reported significant associations between reporting stigma and having CD4 counts of less than 200….” Great to read this sentence as solid evidence to state the seriousness of the problem. However, the authors need to note whether the stigma mentioned here includes the internal stigma.
- In the first paragraph, the authors mentioned stigma in Malawi then moved to global issues in the second paragraph. Again, in the third paragraph, the authors discussed the HIV stigma in Malawi. Following this paragraph, the authors explained about stigma. The authors need to revise the outline of the introduction part to make a better flow.

**METHODS**

- State the reporting guideline used by the authors.
- Most qualitative reporting guidelines (i.e., COREQ) require an explanation related to the credentials of the researchers who collected the data (FGDs and/or IDIs). I could read of it in the part of IDI, but no information for the FGDs. Also, who did the analysis (not only the initials); gender, and credentials? Please explain more, also the statement of “….experienced qualitative researcher” and “…by a team of skilled qualitative research assistants” (i.e., qualitative course, research, years?).
- I prefer the use of “Participants” section instead of “Sample size” due to the quantitative sound of the sub-title (also in table 1).
- “Data collection tools (KII and FGD guides) were translated into local languages (Chichewa and Chitumbuka) and back into English; piloted to check their reliability and validity.”

How did the authors check the reliability and validity of the data collection tools (KII and FGD guides)?

- How did the authors develop the IDI and FGD guidelines?
- Who did the translation into English of the transcripts? How did the authors ensure the validity of the translation?
- Aside from methodology and participant triangulation, are there any other triangulation approaches used by the authors?
- The third sentence of “Participant recruitment and data collection” is too long. Separate into more sentences or use (1), …. (2)…. etc., for a better read.
- Analytical approach: what qualitative data analysis was used by the authors? (thematic?)
- How did the team decide on the data saturation? (i.e., peer debriefing?).
- Time of data collection?

**FINDINGS**

- It would be helpful if the authors provided a table or a narrative way to describe the characteristics of the participants (i.e., gender, age, etc.).
- The authors may provide the codes, categories, sub-themes, and themes developed from the study. A coding tree or table will be beneficial to understand the process.
- The objective of this study was “We explore the **manifestations**, **drivers,** and **impacts** of HIV internalized stigma among people living with HIV in Malawi. Understanding experiences of internalized stigma is the first step to determine befitting mitigation options broadly”.

I can relate the first theme of “perceived sub-groups vulnerable to internalized HIV stigma” to answer the study's purposes. But the second and third themes need to restructure as the article only repeated the study objectives.

I.e. (1) objective: “Understanding **experiences of**…”. Theme: “**Experiences of**

internal (both feelings and manifestations) stigma among people living with HIV”.

(2) Objective: “**impacts of** HIV internalized stigma…”. Theme: “**Effects of**

internalised stigma…”. A coding tree will help the readers understand how these themes developed.

**DISCUSSION**

- “A peer-supporter gives us advice on how we can live happily with HIV”. The

manifestations and coping mechanisms of internalised HIV stigma among people living with HIV in Malawi.

After reading the whole paper, I did not understand why the authors stated this quote in the title. If this quote is highlighted in the title, it should be the “heart” of the findings and discussion parts. Moreover, in the title authors mentioned “The manifestations and coping mechanisms”. Did this quote represent the manifestations and coping mechanisms?

- Strength and limitations? i.e., as the authors did not conduct the member checking.
