# Supplementary material for: Lived experiences of people living with HIV—A qualitative exploration on the manifestation, drivers, and effects of internalized HIV stigma within the Malawian context
Source: PLoS One. 2023 Apr 27;18(4):e0284195. doi: 10.1371/journal.pone.0284195 (PMC10138249; doi:10.1371/journal.pone.0284195)
Supplement: Attachment — Submitted filename: Comments to authors.docx [file pone.0284195.s004.docx]

**Comments to the authors**

**Introduction**

Lines 1-2: “HIV stigma remains pervasive at each point of the HIV care continuum and is profoundly ingrained within social, economic, cultural and historical contexts”

- Use and cite the following current resources to support this statement:

[Psychological and social impact of HIV on women living with HIV and their families in low-and middle-income Asian countries: A systematic search and critical review](https://scholar.google.com.au/citations?view_op=view_citation&hl=en&user=K7SUZ1YAAAAJ&sortby=pubdate&citation_for_view=K7SUZ1YAAAAJ:maZDTaKrznsC).

International Journal of Environmental Research and Public Health 19 (11), 6668. <https://www.mdpi.com/1660-4601/19/11/6668>

[Stigma and discrimination towards people living with HIV in the context of families, communities, and healthcare settings: a qualitative study in Indonesia](https://scholar.google.com.au/citations?view_op=view_citation&hl=en&user=K7SUZ1YAAAAJ&citation_for_view=K7SUZ1YAAAAJ:dhFuZR0502QC). International journal of environmental research and public health 18 (10), 5424. <https://www.mdpi.com/1660-4601/18/10/5424>

Second paragraph: “Globally, the 2020 UNAIDS Global AIDS Update observed that HIV stigma persist to obstruct people living with HIV from making use of HIV related services and that discriminatory attitudes towards people living with HIV continue to be prevalent in many countries with the available legal systems unable to provide protection (11).”

- Use and cite the following current resources to support this statement:

[HIV stigma and moral judgement: qualitative exploration of the experiences of HIV stigma and discrimination among married men living with HIV in Yogyakarta](https://scholar.google.com.au/citations?view_op=view_citation&hl=en&user=K7SUZ1YAAAAJ&citation_for_view=K7SUZ1YAAAAJ:0EnyYjriUFMC). International Journal of Environmental Research and Public Health 17 (2), 636. <https://www.mdpi.com/1660-4601/17/2/636>

[HIV stigma and discrimination: perspectives and personal experiences of healthcare providers in Yogyakarta and Belu, Indonesia](https://scholar.google.com.au/citations?view_op=view_citation&hl=en&user=K7SUZ1YAAAAJ&citation_for_view=K7SUZ1YAAAAJ:4DMP91E08xMC). Frontiers in medicine 8, 625787. <https://www.frontiersin.org/articles/10.3389/fmed.2021.625787/full>

Fifth paragraph: “The internalisation of stigma have been reported to affect quality of life and health outcomes including mental health issues such as anxiety and depression (20), access to HIV-related services (21), adherence to antiretroviral treatment (ART) (22) and willingness to disclose (23).”

- Use and cite the following current resource to support this statement:

[Risk Factors and the Impact of HIV among Women Living with HIV and Their Families in Yogyakarta and Belu District, Indonesia](https://scholar.google.com.au/citations?view_op=view_citation&hl=en&user=K7SUZ1YAAAAJ&sortby=pubdate&citation_for_view=K7SUZ1YAAAAJ:k_IJM867U9cC). PhD Disertation, Flinders University, Adelaide, Australia. (Look at section 5.3.1; 5.4; 6.3.1; 6.4 and chap 7). <https://flex.flinders.edu.au/file/eac1a04e-a24b-4888-ace8-50f06805069e/1/Fauk%20Thesis2022.pdf>

Sixth Paragraph: “Despite the growing debate on getting to the heart of HIV stigma (7,25), researchers have traditionally focused on overt forms of stigma (26,27) without paying attention to internalised forms of stigma that are subtle and operates under the surface. We explored the manifestations, drivers and impacts of HIV internalised stigma among people living with HIV in Malawi.”

- Please look at the section of the dissertation above and revised this gap because there have been many studies exploring internalised stigma.

**Analytical approach**

The authors described the data analysis process they have undertaken, but the steps are not clear to follow. I strongly suggest authors to consult data analysis section of the following resources and cite them to support their description data analysis:

- Structural, Personal and Socioenvironmental Determinants of HIV Transmission among Transgender Women in Indonesia. Int. J. Environ. Res. Public Health **2021**, 18, 5814. <https://doi.org/10.3390/ijerph18115814>
- [Risk Factors and the Impact of HIV among Women Living with HIV and Their Families in Yogyakarta and Belu District, Indonesia](https://scholar.google.com.au/citations?view_op=view_citation&hl=en&user=K7SUZ1YAAAAJ&sortby=pubdate&citation_for_view=K7SUZ1YAAAAJ:k_IJM867U9cC). (Data analysis section: Page 109-114). <https://flex.flinders.edu.au/file/eac1a04e-a24b-4888-ace8-50f06805069e/1/Fauk%20Thesis2022.pdf>

**Reference**

Please revise your reference, look at the journal guidelines about referencing style. There are many mistakes in the references.
