# Supplementary material for: Lived experiences of people living with HIV—A qualitative exploration on the manifestation, drivers, and effects of internalized HIV stigma within the Malawian context
Source: PLoS One. 2023 Apr 27;18(4):e0284195. doi: 10.1371/journal.pone.0284195 (PMC10138249; doi:10.1371/journal.pone.0284195)
Supplement: Attachment — Submitted filename: Rebuttal letter.docx [file pone.0284195.s005.docx]

Moses Kumwenda,

Malawi Liverpool Wellcome Trust,

P.O. Box 30096,

Blantyre 3

Malawi

20^rd^ February 2023

Dear PLoS ONE

**RE: PONE-D-22-27597 Lived experiences of people living with HIV - A qualitative exploration on the manifestation, drivers and effects of internalised HIV stigma within the Malawian context (Revised Title).**

Thank you very much for reviewing our manuscript. We appreciate the comments the reviewers to this manuscript as well as the opportunity given to us to address them. We have taken on board feedback from the editor and reviewers and have revised the manuscript accordingly. In a rebuttal letter, we have included find point-by-point responses to the comments and suggestions made.

In this submission of a revised manuscript, we have included the following documents:

- A rebuttal letter that responds to each point raised by the academic editor and reviewer(s) in a file marked 'Response to Reviewers'.
- A marked-up copy of your manuscript that highlights changes made to the original version in a file marked 'Revised Manuscript with Track Changes'.
- An unmarked version of your revised paper without tracked changes in a file marked 'Manuscript'.

We hope that you will find responses satisfactory.

Kind regards,

Moses Kumwenda (PhD)

On behalf of co-authors

**Journal Requirements:**

When submitting your revision, we need you to address these additional requirements.

1. Please ensure that your manuscript meets PLOS ONE's style requirements, including those for file naming. The PLOS ONE style templates can be found at 
[https://journals.plos.org/plosone/s/file?id=wjVg/PLOSOne_formatting_sample_main_body.pdf](https://journals.plos.org/plosone/s/file?id=wjVg/PLOSOne_formatting_sample_main_body.pdf#inbox/_blank) and 
[https://journals.plos.org/plosone/s/file?id=ba62/PLOSOne_formatting_sample_title_authors_affiliations.pdf](https://journals.plos.org/plosone/s/file?id=ba62/PLOSOne_formatting_sample_title_authors_affiliations.pdf#inbox/_blank)

**Response:** The Title page including title, authors, affiliation and the main body of the manuscript have been formatted according to PLOSONE requirements.

2. Please include a complete copy of PLOS’ questionnaire on inclusivity in global research in your revised manuscript. Our policy for research in this area aims to improve transparency in the reporting of research performed outside of researchers’ own country or community. The policy applies to researchers who have travelled to a different country to conduct research, research with Indigenous populations or their lands, and research on cultural artefacts. The questionnaire can also be requested at the journal’s discretion for any other submissions, even if these conditions are not met.  Please find more information on the policy and a link to download a blank copy of the questionnaire here: [https://journals.plos.org/plosone/s/best-practices-in-research-reporting](https://journals.plos.org/plosone/s/best-practices-in-research-reporting#inbox/_blank). Please upload a completed version of your questionnaire as Supporting Information when you resubmit your manuscript.

**Response:** We have uploaded our data collection guides as supporting information.

3. You indicated that you had ethical approval for your study. Please clarify whether minors (participants under the age of 18 years) were included in this study. If yes, in your Methods section, please ensure you have also stated whether you obtained consent from parents or guardians of the minors included in the study or whether the research ethics committee or IRB specifically waived the need for their consent.

**Response:** Information of ethical approval for the study has been provided in a sub-section ‘Ethics Considerations’ in the methods section of the main body. We have indicated that study ethics clearance was obtained from the Malawi National Commission of Science and Technology (NCST) through its Committee on Social Science and Humanities (Reference No. P.02/21/554). No participant below 18 years was included in the study. We have clarified that adults (18 years and above) were included in the study in the first sentence of Paragraph 5 of the Design and study setting sub-section. We made sure that the youth included in the study were between 18 and 24 years of age. As such, ethical requirements for minors were not applicable in this study.

4. In your Data Availability statement, you have not specified where the minimal data set underlying the results described in your manuscript can be found. PLOS defines a study's minimal data set as the underlying data used to reach the conclusions drawn in the manuscript and any additional data required to replicate the reported study findings in their entirety. All PLOS journals require that the minimal data set be made fully available. For more information about our data policy, please see [http://journals.plos.org/plosone/s/data-availability](http://journals.plos.org/plosone/s/data-availability#inbox/_blank).

Upon re-submitting your revised manuscript, please upload your study’s minimal underlying data set as either Supporting Information files or to a stable, public repository and include the relevant URLs, DOIs, or accession numbers within your revised cover letter. For a list of acceptable repositories, please see [http://journals.plos.org/plosone/s/data-availability#loc-recommended-repositories](http://journals.plos.org/plosone/s/data-availability#inbox/_blank). Any potentially identifying patient information must be fully anonymized.

Important: If there are ethical or legal restrictions to sharing your data publicly, please explain these restrictions in detail. Please see our guidelines for more information on what we consider unacceptable restrictions to publicly sharing data: [http://journals.plos.org/plosone/s/data-availability#loc-unacceptable-data-access-restrictions](http://journals.plos.org/plosone/s/data-availability#inbox/_blank). Note that it is not acceptable for the authors to be the sole named individuals responsible for ensuring data access.

We will update your Data Availability statement to reflect the information you provide in your cover letter.

**Response:** The minimal data set underlying the results described in this manuscript can be found at the Qualitative Data Repository (<https://doi.org/10.5064/F6UJUEP3>). We have also highlighted this in our cover letter.)

4. We note that you have stated that you will provide repository information for your data at acceptance. Should your manuscript be accepted for publication, we will hold it until you provide the relevant accession numbers or DOIs necessary to access your data. If you wish to make changes to your Data Availability statement, please describe these changes in your cover letter and we will update your Data Availability statement to reflect the information you provide.

**Response:** Data availability statement has been revised. The minimal data set underlying the results described in this manuscript can now be found at the Qualitative Data Repository (<https://doi.org/10.5064/F6UJUEP3>).

5. Please amend either the abstract on the online submission form (via Edit Submission) or the abstract in the manuscript so that they are identical.

**Response:** We have edited the abstract in the manuscript so that it is identical to that of the online submission form.

6. Please include your full ethics statement in the ‘Methods’ section of your manuscript file. In your statement, please include the full name of the IRB or ethics committee who approved or waived your study, as well as whether or not you obtained informed written or verbal consent. If consent was waived for your study, please include this information in your statement as well.

**Response:** We have indicated in the methods section that ethics clearance was obtained from the Malawi National Commission of Science and Technology (NCST) through its Committee on Social Science and Humanities (Reference No. P.02/21/554). This information is contained in a section on ethical considerations.

8. Please include captions for your Supporting Information files at the end of your manuscript, and update any in-text citations to match accordingly. Please see our Supporting Information guidelines for more information: [http://journals.plos.org/plosone/s/supporting-information](http://journals.plos.org/plosone/s/supporting-information#inbox/_blank). 

**Response:**  We have included captions for the supporting information files and have referenced these accordingly in the main test of the manuscript.

**Additional Editor Comments:**

**Introduction**

Lines 1-2: “HIV stigma remains pervasive at each point of the HIV care continuum and is profoundly ingrained within social, economic, cultural and historical contexts”

- Use and cite the following current resources to support this statement:

[Psychological and social impact of HIV on women living with HIV and their families in low-and middle-income Asian countries: A systematic search and critical review](https://scholar.google.com.au/citations?view_op=view_citation&hl=en&user=K7SUZ1YAAAAJ&sortby=pubdate&citation_for_view=K7SUZ1YAAAAJ:maZDTaKrznsC#inbox/_blank). International Journal of Environmental Research and Public Health 19 (11), 6668. [https://www.mdpi.com/1660-4601/19/11/6668](https://www.mdpi.com/1660-4601/19/11/6668#inbox/_blank)

[Stigma and discrimination towards people living with HIV in the context of families, communities, and healthcare settings: a qualitative study in Indonesia](https://scholar.google.com.au/citations?view_op=view_citation&hl=en&user=K7SUZ1YAAAAJ&citation_for_view=K7SUZ1YAAAAJ:dhFuZR0502QC#inbox/_blank). International journal of environmental research and public health 18 (10), 5424. [https://www.mdpi.com/1660-4601/18/10/5424](https://www.mdpi.com/1660-4601/18/10/5424#inbox/_blank)

**Response:** We have used the suggested current sources as suggested by the reviewers.

Second paragraph: “Globally, the 2020 UNAIDS Global AIDS Update observed that HIV stigma persist to obstruct people living with HIV from making use of HIV related services and that discriminatory attitudes towards people living with HIV continue to be prevalent in many countries with the available legal systems unable to provide protection (11).”

- Use and cite the following current resources to support this statement:

[HIV stigma and moral judgement: qualitative exploration of the experiences of HIV stigma and discrimination among married men living with HIV in Yogyakarta](https://scholar.google.com.au/citations?view_op=view_citation&hl=en&user=K7SUZ1YAAAAJ&citation_for_view=K7SUZ1YAAAAJ:0EnyYjriUFMC#inbox/_blank). International Journal of Environmental Research and Public Health 17 (2), 636. [https://www.mdpi.com/1660-4601/17/2/636](https://www.mdpi.com/1660-4601/17/2/636#inbox/_blank)

[HIV stigma and discrimination: perspectives and personal experiences of healthcare providers in Yogyakarta and Belu, Indonesia](https://scholar.google.com.au/citations?view_op=view_citation&hl=en&user=K7SUZ1YAAAAJ&citation_for_view=K7SUZ1YAAAAJ:4DMP91E08xMC#inbox/_blank). Frontiers in medicine 8, 625787. [https://www.frontiersin.org/articles/10.3389/fmed.2021.625787/full](https://www.frontiersin.org/articles/10.3389/fmed.2021.625787/full#inbox/_blank)

**Response:** In addition to a source previously included in the manuscript, we have used the suggested current sources as suggested by the reviewers.

Fifth paragraph: “The internalisation of stigma have been reported to affect quality of life and health outcomes including mental health issues such as anxiety and depression (20), access to HIV-related services (21), adherence to antiretroviral treatment (ART) (22) and willingness to disclose (23).”

- Use and cite the following current resource to support this statement:

[Risk Factors and the Impact of HIV among Women Living with HIV and Their Families in Yogyakarta and Belu District, Indonesia](https://scholar.google.com.au/citations?view_op=view_citation&hl=en&user=K7SUZ1YAAAAJ&sortby=pubdate&citation_for_view=K7SUZ1YAAAAJ:k_IJM867U9cC#inbox/_blank). PhD Disertation, Flinders University, Adelaide, Australia. (Look at section 5.3.1; 5.4; 6.3.1; 6.4 and chap 7). [https://flex.flinders.edu.au/file/eac1a04e-a24b-4888-ace8-50f06805069e/1/Fauk%20Thesis2022.pdf](https://flex.flinders.edu.au/file/eac1a04e-a24b-4888-ace8-50f06805069e/1/Fauk%20Thesis2022.pdf#inbox/_blank)

**Response:** We have used the suggested current sources as suggested.

Sixth Paragraph: “Despite the growing debate on getting to the heart of HIV stigma (7,25), researchers have traditionally focused on overt forms of stigma (26,27) without paying attention to internalised forms of stigma that are subtle and operates under the surface. We explored the manifestations, drivers and impacts of HIV internalised stigma among people living with HIV in Malawi.”

- Please look at the section of the dissertation above and revised this gap because there have been many studies exploring internalised stigma.

**Response:** We have revised the sentence to reflect the growing literature on internalized stigma as observed by our reviewer.

**Analytical approach**

The authors described the data analysis process they have undertaken, but the steps are not clear to follow. I strongly suggest authors to consult data analysis section of the following resources and cite them to support their description data analysis:

- Structural, Personal and Socioenvironmental Determinants of HIV Transmission among Transgender Women in Indonesia. Int. J. Environ. Res. Public Health **2021**, 18, 5814. [https://doi.org/10.3390/ijerph18115814](https://doi.org/10.3390/ijerph18115814#inbox/_blank)
- [Risk Factors and the Impact of HIV among Women Living with HIV and Their Families in Yogyakarta and Belu District, Indonesia](https://scholar.google.com.au/citations?view_op=view_citation&hl=en&user=K7SUZ1YAAAAJ&sortby=pubdate&citation_for_view=K7SUZ1YAAAAJ:k_IJM867U9cC#inbox/_blank). (Data analysis section: Page 109-114). [https://flex.flinders.edu.au/file/eac1a04e-a24b-4888-ace8-50f06805069e/1/Fauk%20Thesis2022.pdf](https://flex.flinders.edu.au/file/eac1a04e-a24b-4888-ace8-50f06805069e/1/Fauk%20Thesis2022.pdf#inbox/_blank)

**Response:** We have revised the section on the analytical approach to demonstrate the various steps taken during our data analysis.

Please revise your reference, look at the journal guidelines about referencing style. There are many mistakes in the references.

**Response:** We have revised the references in accordance to journal guide lines.


**Reviewers' comments:**

Reviewer's Responses to Questions

**Comments to the Author**

1. Is the manuscript technically sound, and do the data support the conclusions?

The manuscript must describe a technically sound piece of scientific research with data that supports the conclusions. Experiments must have been conducted rigorously, with appropriate controls, replication, and sample sizes. The conclusions must be drawn appropriately based on the data presented.

Reviewer #1: Partly

Reviewer #2: Yes

**Response:** We have strengthened our conclusion by drawing much from the data.

2. Has the statistical analysis been performed appropriately and rigorously?

Reviewer #1: N/A

Reviewer #2: N/A

3. Have the authors made all data underlying the findings in their manuscript fully available?

The [PLOS Data policy](http://www.plosone.org/static/policies.action#inbox/_blank) requires authors to make all data underlying the findings described in their manuscript fully available without restriction, with rare exception (please refer to the Data Availability Statement in the manuscript PDF file). The data should be provided as part of the manuscript or its supporting information, or deposited to a public repository. For example, in addition to summary statistics, the data points behind means, medians and variance measures should be available. If there are restrictions on publicly sharing data—e.g. participant privacy or use of data from a third party—those must be specified.

Reviewer #1: No

Reviewer #2: Yes

**Response:** The minimal data set underlying the results described in this manuscript can now be found at the Qualitative Data Repository (<https://doi.org/10.5064/F6UJUEP3>).

4. Is the manuscript presented in an intelligible fashion and written in standard English?

PLOS ONE does not copyedit accepted manuscripts, so the language in submitted articles must be clear, correct, and unambiguous. Any typographical or grammatical errors should be corrected at revision, so please note any specific errors here.

Reviewer #1: Yes

Reviewer #2: Yes

5. Review Comments to the Author

Please use the space provided to explain your answers to the questions above. You may also include additional comments for the author, including concerns about dual publication, research ethics, or publication ethics. (Please upload your review as an attachment if it exceeds 20,000 characters)

**Reviewer #1: Comments to the Authors**

Dear authors,
Thank you for the chance to offer feedback. I thoroughly enjoyed your article and believe that paper is worth pursuing.
I have offered some feedback below. I hope you find my comments/suggestions helpful.

INTRODUCTION
- “More than a third of people living with HIV (PLHIV) report having been physically assaulted…” was written/reported in 2012.
As this is a prevalence report, better to provide more recent data/references.

**Response:** We included more recent references and have revised the sentence accordingly.

- “A recent systematic review and meta-analysis reported significant associations between reporting stigma and having CD4 counts of less than 200….” Great to read this sentence as solid evidence to state the seriousness of the problem. However, the authors need to note whether the stigma mentioned here includes the internal stigma.

**Response:** Yes, the stigma mentioned in the sentence include internal stigma. Authors of the paper investigated many dimensions of stigma including internalized stigma described in the paper as self-image, shame, internalized stigma etc. We have revised the sentence to demonstrate that internalized stigma is included.

- In the first paragraph, the authors mentioned stigma in Malawi then moved to global issues in the second paragraph. Again, in the third paragraph, the authors discussed the HIV stigma in Malawi. Following this paragraph, the authors explained about stigma. The authors need to revise the outline of the introduction part to make a better flow.

**Response:** We have revised the outline of the introduction section to improve the flow of the paper.

METHODS
- State the reporting guideline used by the authors.

**Response:** We have mentioned COREQ as a framework that guided our reporting.

Most qualitative reporting guidelines (i.e., COREQ) require an explanation related to the credentials of the researchers who collected the data (FGDs and/or IDIs). I could read of it in the part of IDI, but no information for the FGDs. Also, who did the analysis (not only the initials); gender, and credentials? Please explain more, also the statement of “….experienced qualitative researcher” and “…by a team of skilled qualitative research assistants” (i.e., qualitative course, research, years?).

**Response:** We have included the credentials of individuals who were involved in data collection and analysis in the methods section.

- I prefer the use of “Participants” section instead of “Sample size” due to the quantitative sound of the sub-title (also in table 1).

**Response:** We have replaced the word ‘sample size’ with ‘participants’ as suggested by the reviewer

- “Data collection tools (KII and FGD guides) were translated into local languages (Chichewa and Chitumbuka) and back into English; piloted to check their reliability and validity.” How did the authors check the reliability and validity of the data collection tools (KII and FGD guides)?

**Response:** The sentence has been revised to clarify the aim of pilot testing data collection tools. The sentence now reads ‘Data collection tools (KII and FGD guides) were translated into local languages (Chichewa and Chitumbuka) and back into English; piloted to check their reliability in capturing data that addressed the research objectives.’ We checked if the tools were capturing the information that we wanted to get in order to answer the research questions.

- How did the authors develop the IDI and FGD guidelines?

**Response:** In the methods section, we have included the following sentence that describes how the data collection guides were developed. “Data collection tools (KII and FGD guides) were developed by MK, DK and MC. The development of the guides was framed by the study research questions which were aligned to the International AIDs Society’s’ (IAS) agenda on “Getting to the Heart of Stigma” and shaped by the Health, Stigma and Discrimination Framework.”

- Who did the translation into English of the transcripts? How did the authors ensure the validity of the translation?

**Response:** Members of the research teams included a mixture of individuals fluent in Chichewa and Chitumbuka languages. Please note that Chichewa is one of the national languages in Malawi and as such, most Malawians understands this language. In terms of translation, Hastings Mwanza translated from English to Tumbuka. Khazgani Nasasara translated from Chichewa to English. David Kamkwamba proofread the Chichewa translation while Maureen Chirwa proofread the Tumbuka translation**.** We hoped that these steps were sufficient to ensure the validity of our data collection tools.

- Aside from methodology and participant triangulation, are there any other triangulation approaches used by the authors?

**Response:** There were no other triangulation done apart from those described in the methods section. However, a comparison of the new codes was done between two coders involved in the coding process.

- The third sentence of “Participant recruitment and data collection” is too long. Separate into more sentences or use (1), …. (2)…. etc., for a better read.

**Response:** The sentence has been separated into two sentences. It now reads: “Participants for the initial sample were purposively selected based on their virtue of being people living with HIV, living with disability and HIV and being national/, district, and/ community stakeholders with roles and responsibilities in the development and implementation of the legal and policy instruments and, guidelines. Additional participant’s eligibility criteria included being involved in the allocation of resources for assisting people living with HIV; healthcare providers involved in interventions designed to mitigate effects of HIV stigma; and those involved in fostering legal redress against HIV-related human rights violations.”

- Analytical approach: what qualitative data analysis was used by the authors? (thematic?)

**Response:** We have clarified this in the analytical approach section. Thematic approach was used to analyse data.

- How did the team decide on the data saturation? (i.e., peer debriefing?).

**Response:** We have clarified this in the analytical approach section that ‘data saturation’ was decided at two levels 1) during data collection and 2) during data analysis. At both levels, peer debriefing was used to make this determination.

- Time of data collection?

**Response:** We have included the data collection period in the methods section of the manuscript (6^th^ to 12^th^ April 2021).

FINDINGS
- It would be helpful if the authors provided a table or a narrative way to describe the characteristics of the participants (i.e., gender, age, etc.).

**Response:** We have provided the characteristics of the participants in Table 2 in the introductory part of the finding section.

- The authors may provide the codes, categories, sub-themes, and themes developed from the study. A coding tree or table will be beneficial to understand the process.

**Response:** A coding tree from NVIVO nodes has been provided as Figure 3. The figure illustrates the codes that were generated and merged to generate themes.

- The objective of this study was “We explore the manifestations, drivers, and impacts of HIV internalized stigma among people living with HIV in Malawi. Understanding experiences of internalized stigma is the first step to determine befitting mitigation options broadly”.
I can relate the first theme of “perceived sub-groups vulnerable to internalized HIV stigma” to answer the study's purposes. But the second and third themes need to restructure as the article only repeated the study objectives.
I.e. (1) objective: “Understanding experiences of…”. Theme: “Experiences of
internal (both feelings and manifestations) stigma among people living with HIV”.
(2) Objective: “impacts of HIV internalized stigma…”. Theme: “Effects of
internalised stigma…”. A coding tree will help the readers understand how these themes developed.

**Response:** We have provided a coding tree generated from NVIVO as Figure 3. We have also included the main aim of the study as described in the study protocol and clarified what this paper attempts to achieve. We have also revised the themes subtitles of the findings section.

DISCUSSION
- “A peer-supporter gives us advice on how we can live happily with HIV”. The
manifestations and coping mechanisms of internalised HIV stigma among people living with HIV in Malawi.
After reading the whole paper, I did not understand why the authors stated this quote in the title. If this quote is highlighted in the title, it should be the “heart” of the findings and discussion parts. Moreover, in the title authors mentioned “The manifestations and coping mechanisms”. Did this quote represent the manifestations and coping mechanisms?

**Response:** We have revised the title of the manuscript. It now reads “Lived experiences of People Living with HIV - A qualitative exploration of the manifestation, drivers and effects of Internalised HIV Stigma within the Malawian context. We hope that the new title is broad to cover the full scope of the manuscript.

- Strength and limitations? i.e., as the authors did not conduct the member checking.

**Response:** We have provided a statement on methodological limitation of the study in the last paragraph of the discussion section.

Reviewer #2: First, I would like to thank and congratulate those who are writing this document, since carrying out a study of the problems that are experienced at an institutional and national level seems to me to be an action of resistance, and at the same time of intelligence , by using platforms such as scientific journals (of privilege) to make these latent problems visible today.
It is for the above that the article is built with highly relevant and valuable content to be published for worldwide. Since the speeches clearly capture traces of realities that are oppressed, excluded, and silenced.

**Response:** We thank our reviewers for the wonderful comment on our manuscript.

Second, details are missing on aspects of the design. How were the participants selected? Why were the areas of the country chosen? Why sex workers? How old are the participants? At no time is the age range mentioned. These are critical methodological details required... can you provide details? Likewise, as a fundamental point, they do not mention from which paradigm or epistemology, approach or perspective they are located to analyze the investigative study.

**Response:** Participants were purposively sampled. Participants were selected by virtue of their being people living with HIV and disability, national, district and community stakeholders with roles and responsibilities in development and implementation of the legal and policy instruments, guidelines; those engaged in allocation of resources for assisting people living with HIV; health care providers involved in interventions which are designed to mitigate the effects of specific internal stigma mechanisms tied to specific health behaviors and outcomes and those involved in facilitating legal redress against human rights violations amongst people living with HIV and disability. Key informant interviews, Focus Group Discussions and life stories were used to gain insights on how people living with HIV and disability stigmatize themselves and how they interpret the efforts on stigma reduction in the existing laws, policies, guidelines and structures/systems. Existing collaboration and networks within the team was used to engage networks of people living with HIV across affected population groups, geographical divide of the disease burden, cultural and ethnic diversity in Malawi to draw the participants.

Finally, can your discussion provide a more detailed discussion on what is the main contribution of your study to the existing literature and what are the implications of your study and how it differs from other studies already done?

**Response:** We have included a discussion of the main contribution and implications of study findings in the second paragraph of the discussion section.

Lastly, what psychosocial intervention proposal can be built to deliver solutions to the problems that have emerged?

**Response:** No psychosocial intervention directly emerged from this study. But as researchers, the findings from this study have helped us to start conducting other studies that have psychosocial interventions for managing common mental disorders such as depression but also internalized HIV stigma. We are currently piloting a Tendai intervention for managing depression among HIV positive women in Blantyre. We have also developed tools for providing information on treatment as prevention in the hope that to normalize this knowledge within our contexts and reduce HIV related self-stigma.

6. PLOS authors have the option to publish the peer review history of their article ([what does this mean?](https://journals.plos.org/plosone/s/editorial-and-peer-review-process#inbox/_blank)). If published, this will include your full peer review and any attached files.


If you choose “no”, your identity will remain anonymous but your review may still be made public.

**Do you want your identity to be public for this peer review?** For information about this choice, including consent withdrawal, please see our [Privacy Policy](https://www.plos.org/privacy-policy#inbox/_blank).

Reviewer #1: No

Reviewer #2: **Yes:** Estefany Castillo Ravanal

[NOTE: If reviewer comments were submitted as an attachment file, they will be attached to this email and accessible via the submission site. Please log into your account, locate the manuscript record, and check for the action link "View Attachments". If this link does not appear, there are no attachment files.]

While revising your submission, please upload your figure files to the Preflight Analysis and Conversion Engine (PACE) digital diagnostic tool, [https://pacev2.apexcovantage.com/](https://pacev2.apexcovantage.com/#inbox/_blank). PACE helps ensure that figures meet PLOS requirements. To use PACE, you must first register as a user. Registration is free. Then, login and navigate to the UPLOAD tab, where you will find detailed instructions on how to use the tool. If you encounter any issues or have any questions when using PACE, please email PLOS at [figures@plos.org](mailto:figures@plos.org#inbox/_blank). Please note that Supporting Information files do not need this step.
